# Technology development for the early detection of plant pests: a framework for assessing Technology Readiness Levels (TRLs) in environmental science

**DOI:** 10.1007/s41348-022-00599-3

**Published:** 2022-06-20

**Authors:** Rehema White, Mariella Marzano, Elena Fesenko, Alan Inman, Glyn Jones, Barbara Agstner, Rick Mumford

**Affiliations:** 1 School of Geography and Sustainable Development, University of St Andrews, Irvine Building, North Street, Fife, KY16 9AL Scotland; 2 Forest Research, Northern Research Station, Roslin, Midlothian EH25 9SY Scotland; 3 Present Address: Food Standards Agency, Foss House, Kings Pool, 1-2 Peasholme Green, York, YO1 7PR England; 4 Fera Science Ltd, National Agri-Food Innovation Campus, Sand Hutton, York, YO41 1LZ England

**Keywords:** Innovation, Tree health, Biosecurity, Co-design, Knowledge implementation, Plant health surveillance

## Abstract

Innovation in environmental fields such as plant health is complex because of unbounded challenges and lack of certainty of commercial uptake. In this paper we present a Technology Readiness Level (TRL) framework, specifically to assist with assessment of technologies to support detection of tree pests and pathogens, but also for wider potential adaptation. Biosecurity can be enhanced by improved early detection of pests and pathogens, but development and deployment of new technologies requires robust scrutiny. We critically analyse the concept, practice and applicability of TRLs. Interviews revealed scientist perspectives during the development process of five novel early plant pest and pathogen detection technologies. A retrospective, collective narrative of one technology from concept to commercial deployment was undertaken. We then developed a calculator tool for assessment of biosecurity TRLs. Our findings illustrate the iterative process of technology development, the challenges in final TRLs of acquiring funding to move from proven success to viable product, inefficiencies created through the need for multiple projects for each technology and the imperative to consider the wider socio-ecological technical landscape, including policy context. End user engagement was particularly valuable at beginning and end of the TRL scale. We conclude that the TRL framework comprises a robust approach to assess technologies in that it facilitates progress tracking, evaluation of success likelihood and identification of opportunities for investment. However, its potential will only be realised for environmental management if it is integrated into the socio-ecological technical landscape and wider discussions regarding knowledge co-production and valuing nature.

**Supplementary Information:**

The online version contains supplementary material available at 10.1007/s41348-022-00599-3.

## Introduction

We face increasing, complex and interlinked environmental challenges at a global level, including biodiversity loss, climate change and pollution (Rockstrom et al. 2009). One concern is the threats posed to the ecological resilience of our trees, woodlands and forests by pests and diseases (hereafter known as pests) (Freer-Smith and Webber 2015; Boyd et al. 2013). Increased threats derive from macro issues such as globalisation, with increased trade and movement of goods and socio-cultural changes in expectations for consumption (Brasier et al. 2008; Webber 2010; Marzano et al. 2017). Pests can be introduced through trade in live plants (Webber 2010; Liebhold et al. 2012) and wood packaging materials (Brokerhoff et al. 2006) but also via biomass and through human recreational activities. Outbreak eradication is most effective during the earliest phases of invasion (Pluess et al. 2012). Currently, most countries rely heavily on trained inspectors to identify pests, mainly via visual inspections. Practical barriers include huge volumes of material for inspection, difficulties in accessing consignments and complexities in detecting cryptic targets. However, the shift towards governance and the diffusion of the border mean that some shipments are also examined at target locations such as plant nurseries, rather than merely at ports of entry, and detection may be required within existing woodlands and forests (Marzano et al. 2018; White et al. 2018). Thus, there is a complex suite of actors and locations involved in detection of tree pests and pathogens within a dynamic socio-ecological system (Dandy et al. 2017).

In order to improve biosecurity for tree health, new tools and approaches are needed to facilitate better surveillance and monitoring systems and to enhance our detection efficiency for both known and unknown threats. There is also a need to extend beyond the traditional inspector-based model of plant health, working with stakeholders to deliver effective biosecurity. These tools will include new scientific concepts and the development of technologies that are fit-for-purpose and meet the needs of regulators (e.g. government), those who are impacted by regulation (e.g. woodland owners and industry) and end-users (e.g. inspectors in the field). Whilst accurate pest identification is a scientific issue, detection is multi-faceted, requiring a focus on the real-world opportunities and obstacles (be they environmental, regulatory, fiscal and social) that can enhance or delay the successful deployment of novel technologies (Callaway 2017). For example, the stakeholder landscape around tree health is complex (Dandy et al. 2017), governance and policy contexts are dynamic (Klapwijk et al. 2016; Marzano et al. 2017) and there are limited resources with which to achieve biosecurity aims (White et al. 2018). Despite these challenges, there has been a recent focus on developing in-field diagnostics, exploring the gap between research and deployment and examining the role of stakeholder engagement in the technology innovation process (Marzano et al. 2018; White et al 2018).

**Fig. 1:**
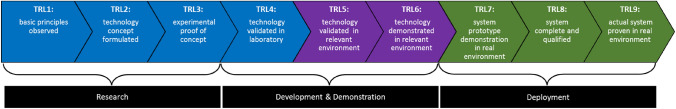
TRL scale (blue-laboratory environment; purple—relevant, i.e. simulated, environment; green-real environment)

The most widely adopted approach for quantifying the maturity and comparing the readiness of different technologies is the Technology Readiness Levels (TRL) framework, although this has not to date been used in biosecurity. Originating from the space industry, TRLs were first developed and used in the 1970s and 1980s by the National Aeronautics and Space Administration (NASA) (Sadin et al. 1989). Originally employed as part of an overall risk assessment process, by the early 1990s TRLs were routinely used within NASA to support technology maturity assessments and comparisons of maturity (Mankins 2009). The first detailed definition of each level, together with examples, were undertaken in 1995, and since their early conception and adoption in the space, aviation and defence industries, TRLs are now used in other sectors, such as health (Mankins 2009; see below). TRLs are based on a nine-point scale that represents activities from research (TRL 1–3) to development and demonstration (TRL 4–6) and production and deployment (TRL 7–9) (Fig. 1).

Technology deployment depends not only on the excellence of the research, but also on application and commercial factors. All technologies face challenges in relation to performance, schedule of development and budget (Mankins 2009). New technologies need to be assessed for design, engineering, manufacturing and end use in real world conditions before any consideration of commercialisation (Heslop et al. 2001; Clausing and Holmes 2010).

Our goal in this study was to evaluate how we might assess the readiness of technologies to be used in the early detection of tree pests and pathogens, and thus enhance technology innovation for environmental management. We sought to address four research questions: What are the challenges and opportunities for the use of TRLs in technology development for the early detection of tree pests and pathogens? How do scientists and stakeholders perceive socio-technological innovation and TRLs across a suite of emerging technologies? How can TRLs be applied across the process of development of a successful, mature technology? What are the wider implications of technology development for tree health, biosecurity and environmental management more widely?

We begin by outlining our methodological approach and methods of data collection. We then outline the concept and potential of the TRL framework. The perceptions of scientists leading the development of five early detection technologies are explored and a retrospective narrative from concept to deployment of one further technology is developed. We present a calculator tool to facilitate TRL assessment in environmental contexts and we conclude with discussion and recommendations.

## Materials and methods

This was a multi-method analysis of technological innovation in the environmental sector. Firstly, we conducted a literature based analysis of the TRL concept. We used the search terms “TRL”, ‘TRLs”, “technology readiness” and “Technology Readiness Level” using both search engines for the academic literature (including Google Scholar and Seeker) and wider platforms for grey literature (Google). We pursued further papers and reports identified in reference lists of initial search finds until we reached saturation. We also explored literatures regarding technological innovation to frame the field and identify related topics. This analysis was not intended to create a complete bibliography, but rather to enable us to present the history, scope and application of the concept.

Secondly, we investigated the perspectives of scientists engaged in technology development as part of a project involved in pest early detection innovation (Mumford et al 2017).^1^ Ten semi-structured interviews were undertaken with scientists (at different research stages) who were researching five promising technologies: volatile organic compounds (VOC), multispectral imaging, air and a separate water-borne spore trapping network linked to next generation sequencing and pheromone traps. All interviews were undertaken by two of the authors (RMW and MM), recorded, professionally transcribed and thematically coded (Bryman 2001). Themes included technology maturation journeys, TRL assessment, challenges for technology application and forms and influences of stakeholder engagement. Early interview data revealed that the process of technology development was long term and cumulative. We also wished to understand how a technology could be assessed across its lifespan, from concept to application. Thirdly, we developed a retrospective narrative of one early detection technology (LAMP: Loop- Mediated Isothermal Amplification; several authors were involved in its development and analysis—EF, AI, GJ and RM). The narrative was developed from personal experiences and modified by wider discussions with other researchers at Fera Science Ltd and potential funders (including the UK Department for Environment, Food and Rural Affairs (Defra)). Fourthly, in order to specifically investigate how the TRL framework might be applied in practice, we developed a calculator, a spreadsheet based tool that could be used for further technologies to illustrate readiness at any one stage and influence resource and strategy decision making. This calculator was piloted using the LAMP technological trajectory with several iterations produced. Due to space constraints, only the final version is shown in this paper.

## The TRL framework

A key purpose of the TRL assessment is to “*reveal the gap between a technology’s maturity and the maturity demanded for successful inclusion in the intended product*” (Graettinger et al 2002:5). If early research and development is poorly planned then subsequent projects, which plan to employ technologies, will suffer from a range of negative impacts, including cost overruns, delays and a steady erosion of initial objectives (Clausing and Holmes 2010). Assessment of readiness permits evidence-based decisions about feasibility. Addressing technology problems only at product development phase can increase costs ten fold, or one hundred- fold if resolution is delayed until after production begins (GAO 2001). Overall, the TRL framework has potential to provide reassurance to researchers and funders that new technologies have a “route to market” and will result in products and services with the appropriate performance criteria which meet end-user requirements (Mankins 2009).

While the TRL framework has been used most for physical products and engineering solutions, the underlying principles behind it are potentially transferrable to other sectors. For example, the TRL framework offers a practical template by which to distinguish between the different stages of research and development (from concept to applied research, into development and ultimately commercialisation) for any technology. However, there are challenges; for example, the specific descriptions of the levels developed in an engineering context lack direct relevance to biology-based technologies and neglect the potential complexities of environmental challenges. Hence, when the TRL approach has been used in other industries or sectors, it has been adapted. As other organisations and industries have embraced TRLs, they have moved them away from the original NASA TRL approach to their own specific definitions and terminologies to make them fit their particular needs (for example, EARTO 2014). Whilst most adaptations remain in high technology contexts (e.g. aviation, defence), there have also been uses in other areas of public interest such as drug assessment by US Department of Health and Human Services (EARTO 2014), wave energy technology development (Weber 2012) and composite recycling (Rybicka et al 2016). As a result, there has been a proliferation of TRL definitions, and with it a shift from the simple and readily understood approach offered by having one single system. In order to address this and ensure a globally harmonised approach to using TRLs, in 2009 the European Space Agency proposed that ISO should develop a common TRL definition, which in 2013 resulted in the publication of ISO standard 16,290 “Definition of Technology Readiness Levels (TRLs) and their criteria of assessment”. Now the ISO TRL scale is a standard in the space sector (ISO 16290:^2013^).

Often TRL assessments have been built into a wider project management toolkit, allowing for the ongoing assessment of a specific technology’s progress. This requires multiple assessments over time, from the beginning: small-scale assessments, involving just local research and development staff, or large, highly formal processes based on a multi-actor approach, including external stakeholders (Britt et al 2008). While evidently more resource intensive, the latter approach is deemed to be the most effective and is the one adopted by, for example, US space and defence agencies. As TRLs are adapted to specific fields, there is increasing use of subjective but expert analyses to undertake assessment of TRLs. An attempt to develop automated approaches that evaluate TRL through text-mining techniques and semantic indexing of relevant documents has also been developed and shown to be effective in certain fields (Britt et al 2008), although its application to specific areas such as pest detection may be limited. To support the evaluation process, several TRL assessment tools, or calculators, have been developed, including those from the United States’ AFRL (Air Force Research Laboratory)^2^ and NASA.^3^ Usually, the calculators are based on a set of questions presented using generic software (e.g. Microsoft Excel) that produces a graphical display of TRLs achieved. These tools are intended to provide a snapshot of technology maturity at a given point in time and support management and resourcing decisions.

In some cases, a Technology Readiness Assessment is undertaken using metrics or matrices other than TRLs (Heslop et al. 2001; Clausing and Holmes 2010). For example, Heslop et al. (2001) identified a four part “cloverleaf” framework in which technology strength, market attractiveness, commercialisation avenues and management support areas were used to offer technology readiness assessments, mainly for individual products. In some situations, it has been considered that the TRL approach alone is not sufficient. In the context of multiple competing technologies being developed, a Technology Performance Level scale for techno-economic performance was used alongside TRLs in a TRL-TPL matrix to assess wave energy technologies (Weber 2012). There has been an attempt to explore the relevance of a TRL 10 that indicates a proven technology as demonstrated through extended operations (e.g. Straub 2015), although this has not yet been formally adopted. A further development has been the suggestion of Systems Readiness Levels to support the integration of multiple technologies (Sauser et al 2006). However, that view of a “system” appears to be more comparable to a physical object (such as a spacecraft) than a socio-ecological system such as that for tree health. The Multi-level Perspective (MLP) approach offers a framing for technological innovation in which innovation dynamics play across three levels: niches, socio-technical regimes and the socio-technical landscape (Geels 2010). Whilst MLP explains dynamic shifts and transitions in innovation and technology uptake, it tends to assume that a technology is static, hence Yakamura et al. (2013) investigated how TRLs might link with the MLP approach. They concluded that a niche innovation (particular technology) cannot progress to high TRLs unless there is market and social feasibility, thus interactions between technology and diverse social factors are critical in determining the success of technologies within society. They showed how a “window of opportunity” within the socio-technical landscape (including market, policy and practice aspects) can permit and promote research and investment in particular technologies. One challenge is that the original TRL scale was based on an assumption that the innovation process is linear (EARTO 2014). In addition, EARTO (2014) notes how the concept of TRLs arose around development of a single technology product that would be integrated with other technologies in a complex “mission”. EARTO (2014) acknowledge the “valley of death” that can be experienced in technology development in Europe, where wider research (often in academia) fails to be commercialised and reach market.

We thus see widespread application of a standardised TRL framework across several sectors, with sector specific modifications. There has been an emphasis on highly engineered outputs but increasing adoption across other areas and a recognition that the success of any technology innovation will also be influenced by the wider socio-technical landscape. We now consider whether the TRL framework would be appropriate for plant biosecurity and other environmental systems.

## Researcher perspectives on early detection technology innovation and TRLs

A three year, multi-institutional, interdisciplinary project both supported early detection technology innovation and deepened engagement and knowledge sharing across the plant health sector (Mumford et al 2017; Marzano et al 2018; White et al 2018). Lead researchers for each of the five technologies were interviewed to explore the socio-technological innovation process and determine what TRL they would assign at different time points.

Researchers stated that continuity was required to successfully develop technologies. However, public sector funding is often awarded over 3–4 year research funding cycles. There can be expectations of a finished product at the end of a short project, but in reality it can take 10–15 years from concept to deployment across multiple funding cycles.

*“…of course all of those technologies [the five project technologies] have actually been developed and are being supported by…previous projects, ongoing projects and potentially now future projects as well…The one thing we've also seen from doing some of this work and looking at how you progress the technology through is that it never seems to be related to a single project that takes something and pushes it through to the end”.*

In addition, achieving the final TRLs: “…*was best furnished with lots of little projects trying to tick off all of these little problems, be they technical, or more about what you are using it for*…”.

Working with a TRL framework could help track progress across multiple projects and offer confidence regarding applicability. One researcher discussed how lack of confidence in one specific technology meant that it was not used in a particular pest outbreak, leading to the loss of many trees:

*“…we could have been able to look at all of the trees and say 'which of these trees contain larvae, and which don't, and we will only cut down the ones that contain larvae'. But the technology was still kind of stuck at TRL 6 or 7, and so they didn't have sufficient confidence that they could use it in contingency response. And so they cut down all of the trees”.*

There was acknowledgement that getting from prototype to the final TRL is often the most difficult part, particularly if this is the first stage at which the technology is presented to potential end users:

“…*getting over the last TRLs is quite surprisingly hard. When you get that close you think 'aah, we've just got to do those experiments and then we are done'. And that's where you then start to get into training and users, for example. And suddenly they're like 'ooh, this isn't quite what we wanted to do', or 'we didn't want that target, we wanted you to look at this target'.* ”

This early detection technologies project supported stakeholder engagement and investigated its influence on socio-technological innovation Researchers discussed the value of engagement especially at low and high TRLs.

“*So we put a part of the budget aside to do some co-design workshops, to try to take a step back …from the technologies and the solutions to say 'what are the problems, what are you trying to solve with detection'? And then can we work our way towards a solution, and then develop the work programme appropriately because… we [scientists] quite often end up sitting in a room and immediately leap to solutions*.”

It was acknowledged that stakeholders might find it easier to interact with a prototype rather than a conceptual idea.

*“You kind of almost need to create technology straw men…and actually at the early stage, you should create something that you could throw out, even if it's kind of virtual, or even if it's a mock-up and then at that point people will engage with it, because until then, you know, they can't deal with concepts…”*

It is not only whether the technology has reached a certain TRL scale but also what to do with a technology prototype that was considered. In relation to spore trapping, researchers felt that the technology had reached TRL 7, but this raised new questions about relevance:

“…*now the question becomes more of one of why would you do it, and what would you do with the information?...it isn't clear, and that will slow it down enormously I think, in terms of understanding what we would do with that data, how we would handle it, is it worth doing, is it worth the expense*?”

Importantly, by reflecting on the development and use of their technologies, researchers found it difficult to assess where they were on the TRL scale. Thus, it was felt that a more objective and universal assessment of TRLs was needed and the team proposed development of a calculator tool that facilitated the readiness assessment decision.

“…*when we first … tried it [TRL framework] without the tool, we found that it was very subjective, and it was very hard for people to… really work out where their technologies sort of lay, so you look at some things you go 'that seems very high, or that seems very low' so this should be a more uniform way of pinning a TRL level to technology*.”

Finally, researchers described an iterative rather than linear process for technology development. Hence, researcher perspectives demonstrated the complexity of assessing TRLs, the messy nature of research, the importance of stakeholder engagement at different stages, additional barriers to uptake, the need for multiple funding cycles and the long time frame over which a technology would be developed. The latter realisation led us to undertake a retrospective analysis of one early detection technology across its whole development trajectory to better understand TRL assessment at different stages.

## Retrospective application of TRLs to technology development process: The GENIE and the LAMP

The development of on-site detection technologies for use in plant health began with lateral flow devices (LFDs) in the mid to late 1990s (Mumford et al. 2016). The need for sensitivity and specificity on site led to newer DNA-based diagnostic methods such as the polymerase chain reaction (PCR). A portable PCR machine (the Cepheid Smart Cycler) could be used outside the laboratory to detect plant pests and pathogens. The UK was the first country to use this platform in the field for plant health (Tomlinson et al*.*, 2005). However, key constraints to deployment included: (a) high costs, (b) complex DNA extraction steps (c) cross-contamination problems. This led to research on other platforms and chemistries, resulting in the selection of a more-robust and suitable chemistry for field use (Loop- Mediated Isothermal Amplification: LAMP) linked with a more-portable, cheaper and user-friendly platform (the Genie machine from Optisense). The Genie and LAMP system reached the point of deployment in mid-2015 with trained inspectors making front-line diagnostic decisions (without recourse to laboratory confirmation) at a licensed facility at Heathrow airport for *Liriomyza spp.* leafminers on plants imported from South America.

Between 2006 and 2015, the Genie and Lamp technologies were developed using around 20 different funding routes. Collaboration with SMEs with an applied market focus and route to commercialisation facilitated the final focus. Funding came primarily from longer-term EU projects plus short- and longer-term funding sources from the UK government. Mapping contribution to TRL progress paints a complex picture as the technology was considered for a number of different applications as it was being developed. Key steps and issues that were specifically taken for Genie and LAMP technology development are highlighted in Table 1. Some steps and projects overlapped or ran in parallel. However, development phases can be approximately aligned with TRL concepts. An initial feasibility study funded by a government department and later match funding by government against external resources were seen to be essential aspects of LAMP technology progression.

**Table 1:** Application of the TRL concept for the development of LAMP technology

TRL	Cluster	General description	LAMP technology example
1	Invention	Basic principles observed: initial translation of basic science into potential new basic principles that can be used in new technologies	Advances in understanding of basic molecular biology
2		Technology concept formulated: potential applications are identified but they are speculative with no or limited analyses to support claims of a new technology	Concept development for iso-thermal DNA amplification with discovery of novel DNA replication enzymes
3	Concept validation	Experimental proof of concept: based on preliminary study, actual research is conducted to assess technical and market feasibility of a new technology	Development of LAMP assays and Genie
4		Technology validation in laboratory: basic technological components are integrated to assess early feasibility by testing in laboratory environment	Testing of a model LAMP assay for plant diagnostic purposes
5	Application development & prototyping	Technology validation in relevant environment: advanced testing and refinement with the focus on specific end-user requirements	Design of LAMP-based test for detecting a specific target in a specific host/matrix
6	Demonstration & technology transfer	Demonstration in relevant environment: fine-tuning of a product / process / test and real sample testing and validation	Bringing and testing of whole diagnostic including sampling, extraction and test
7		Demonstration in real environment: a product / process / test is tested by end-users	End-users (APHA, Diagnosticians) test LAMP in the field*
8	Operational validation	System complete and qualified: a new technology performs to end-user specification and qualified through test and demonstration	Extensive field* validation & refinement by PHSI (on site technologies) or diagnosticians (laboratory-based technologies)
9	Deployment	Actual system proven in real environment: a product / process / test is fully operational and competitive	Diagnostic hand over to PHSI for routine use with support in place, e.g. access to reagents, tech support, etc

^*^Field means the actual use by end-users under normal conditions (on-site, for on-site technologies; or in diagnostic laboratory for laboratory-based technologies), i.e. a routine use

Although the Genie and its isothermal LAMP technology are part of an evolution in on-site diagnostics, there is also a range of novel impacts and benefits for plant health, both now and potentially in the future (e.g. paving the way for future detection platforms and chemistries that address the next evolution of operation need). The Genie platform reached potential deployment within three years, but actual deployment was delayed for a further three years due to lack of governance and decision-making processes and because the large number of funding sources created inefficiencies along the pipeline. This highlights the importance of early and effective engagement and long-term capacity building with policy-makers, frontline regulators, industry and other stakeholders; ensuring that ‘solutions’ are fit-for-purpose, there are routes to commercialisation and they offer a genuine cost benefit. In these contexts, using a TRL framework can inform investment decisions and help visualise roadmaps to deployment as well as ensuring that there is continuity provided by a lead organisation or developer.

This narrative reveals the complexity of a technology evolution over almost 20 years; how technologies can transfer across sectors in applicability; how multiple funded projects are required to achieve deployment; how final innovation stages can be delayed; the importance of cost benefit analyses; and a trend towards field based diagnostics by local users rather than sample collection and identification in the laboratory by scientists, requiring greater stakeholder engagement in development.

## The development of a TRL calculator for early pest detection technologies

Difficulties in subjectively assessing TRL stages were identified both through interviews with scientists and LAMP narrative creation. To provide a more robust assessment we thus developed a TRL assessment tool customised for use with diagnostic (or indeed other life science) research and development applications. This tool was based upon the framework of an Excel Workbook-based calculator, which had been designed and made freely available for use on the Internet by the New York State Energy Research and Development Authority (NYSERDA).^4^ The original NYSERDA TRL calculator was based on the systems developed by both NASA and US DOE and was targeted at the cleantech industry. It posed a series of closed “yes” and “no” questions, then calculated an appropriate TRL. The questions were grouped into the seven core sections: (1) General Summary of Technology Readiness; (2) Market and Customer Need; (3) Design and Development; (4) Integration; (5) Testing and Validation; (6) Environmental and Safety and (7) Manufacturing and Scale-Up; with each section highlighting the complexity of the technology development process and roles of different actors. The questions in the original calculator were very much focused on engineering and manufacturing issues (e.g. “*detailed design drawings completed to support engineering-scale system*”) and thus were not always directly relevant to biosecurity scenarios. Therefore, the questions were reformulated while maintaining the overall standardised TRL framework. The draft calculator was piloted after mock assessments of relevant technologies and adjustments were made: the overall number of questions was reduced, a multi-actor approach was introduced, including stakeholder and end-user considerations, and the questions were regrouped into three broad sections: (1) Technology Development; (2) Technology and Deployment; (3) Business Development (Table 2).

**Table 2:** TRL calculator questions used to assess readiness in plant health research

TRL	Answer	TRL	Answer
Technology Development		4	Have you undertaken an assessment to identify risks to end-users?
1	Have the basic scientific principles, which form the foundation of a new technology, been confirmed or reported elsewhere?	6	Has the safety of the technology been assessed and confirmed?
2	Has your technology/concept been described in sufficient detail to define future applications?	7	Has your technology been shown to be safe to use in the environment?
2	Have initial performance predictions of your technology been made?	7	Have test partners been identified?
2	Have publications or other references that outline a new technology been evaluated?	8	Has an aftercare strategy (maintenance, troubleshooting guide or failure analysis document, support plan) been developed?
3	Has a prospective application been specified in sufficient detail to identify all necessary technological elements?	9	Have all safety documents been completed?
3	Have predicted performances of all individual technology components been confirmed by repeated, rigorous and verifiable experiments or simulations in a laboratory environment?	9	Have all necessary end-user documents been developed and made available?
3	Has it been shown that all technology components will work together?	Business Planning	
4	Have you described real-world deployment in detail?	1	Have you outlined the new capabilities which might result from your new technology?
4	Has your technology (including components) been investigated in a laboratory environment with the anticipated results?	1	Have you identified where the capability could be used?
5	Has a detailed process which leads from a demonstration to an application been established?	2	Has the potential of the concept/technology to end-user groups been illustrated?
5	Has a laboratory environment been modified to approximate a real environment (i.e. relevant environment), including the development of a testing protocol?	2	Has a qualitative assessment of risk to the development of your technology been carried out?
5	Have demonstrations in a relevant environment—including individual and integrated testing of all key elements—produced anticipated results?	3	Have you asked end-users if the technology is fit for purpose?
5	Are test results in a relevant environment consistent with technical and economic viability?	3	Has an assessment of market opportunities been carried out?
6	Is your technology described sufficiently to finalise a deployment strategy?	3	Have the preliminary costs of your technology been estimated?
6	Have all relevant test issues (including scaling up) been investigated and resolved?	3	Has a strategy to identify and protect intellectual property been developed?
6	Has the operational performance (e.g. sensitivity, selectivity, etc.) of your technology been fully optimised in a relevant environment?	4	Have the needs for international or domestic patent protection been assessed?
7	Has it been shown, through repeated, rigorous and verifiable demonstrations, that your technology can function in a real environment?	4	Has the performance of your technology been discussed with end-users?
7	Has your technology performance been tested under critical/extreme conditions?	5	Has an intellectual property protection approach been implemented?
7	Have you developed a deployment plan?	5	Has end-user feedback been received to establish a final specification of your technology (agreement of performance needs etc.)?
8	Has your technology received satisfactory feedback after being tested by an end-user in a real environment?	6	Have preliminary price estimates been prepared?
8	Have all verification, validation, and accreditation tests been completed?	6	Has a business case been drafted for the communication with prospective end-users?
9	Has your technology been fully described in terms of conventional use and integration into customer systems?	7	Have patent claims, if applicable, been drafted?
9	Has it been shown that your technology operates at levels of performance, cost, quality, reliability, etc. which have been specified in the business case?	7	Have you done soft market testing?
Technology deployment		8	Have final cost estimates of a new technology been made?
2	Has a preliminary technology development plan to reach deployment been outlined?	8	Is an agreement with at least one paying end-user (i.e. innovator or early adopter) in place?
3	Have provisional arrangements been made for real-life testing?	9	Has a patent application / licence (if applicable) been submitted?
3	Have you identified any hazards associated with your technology?	9	Has a business case been finalised and verified?

**Fig. 2:**
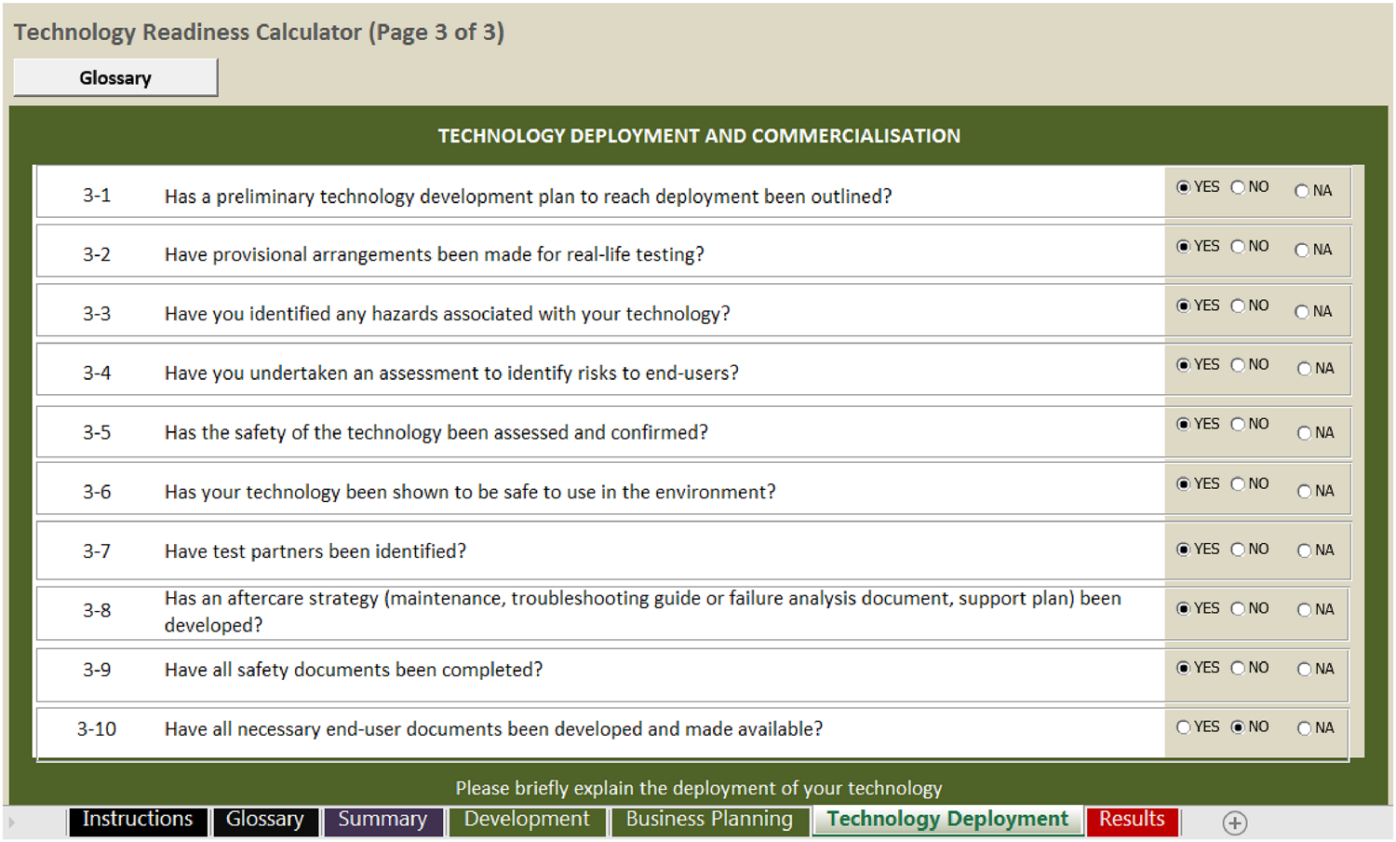
Example of TRL calculator tool questions in screenshot from generic software platform

The calculator was then piloted by professionals involved in the technology development, including scientists and end users (inspectors). Additional options of “not known” and “not applicable” were added to some closed questions. Specific questions assessed readiness in terms of the market and also policy frameworks (Table 2 and Fig. 2). The assessment trial highlighted that different stakeholders have varying perceptions of technology maturity and robust assessments are best made by multiple stakeholders for a given technology.

Finally, the calculator was used to describe the overall innovation trajectory of LAMP. This demonstrated that progress is not always a linear process. For example, sometimes it had to be taken back to a lower readiness level, in order for it to be redesigned, so it could fulfil the requirements of end-users or comply with specific regulations. It also showed that a technology can be at a high TRL overall, yet without full completion of all the perceived lower TRLs. For example, research and development can be fully developed and integrated (and hence technically functional), but if end-users are unwilling to pay extra for a new technology or a regulation framework restricts its application, then it will not reach full deployment at TRL 9. The ability to assess the completion of each TRL was thus used to identify bottlenecks, target investments and appraise overall feasibility.

## Discussion

TRL frameworks have been successfully applied in a range of sectors to ensure that technological innovation addresses requirements for performance, budget and schedule (Mankin 2009). Our study has demonstrated that the TRL framework can help identify and support the development of appropriate technologies for plant health and biosecurity, and potentially other environmental areas. The use of TRLs can build confidence in the readiness of a technology for deployment, especially in legislated or crisis situations. Challenges exist with the subjective application of TRLs by scientists and it was found that a calculator tool, such as the one we developed, combined with assessment by additional non-researcher stakeholders, led to better defined TRL assessment. Within biosecurity, this is a pragmatic response to the challenges of identifying a particular TRL during development either through a subjective rapid appraisal or through a costly expert analysis (Britt et al. 2008).

The use of the TRL framework in this research not only illustrated how and why we might better assess technology readiness, but also highlighted interesting and useful aspects of technology innovation in the environmental sector. Firstly, analysis of TRLs by scientists and the retrospective LAMP TRL journey demonstrated that the process of innovation is iterative, with some technologies retreating back down the TRL scale in order to solve a problem before progressing further again. Original TRL frameworks assumed linearity of development, although EARTO (2014) suggested some nonlinearity may occur. We thus propose that assumption of some iteration be assumed as standard in technology innovation; in practice this could be addressed by including some feedback loops, as appropriate, in TRL frameworks.

Secondly, TRL assessments enabled us to realise the long time frame to move from concept to deployment. The approximately 10–15 year journey of LAMP was not atypical from scientist perspectives. Resource for innovation of this technology was derived across multiple projects usually in 3–4 year cycles and from approximately 20 sources, but this ‘stop-start’ across projects was seen to create inefficiencies in the TRL progression. LAMP analysis also demonstrated that there could be a three year delay in finding funding to take a proven concept to a field based product. This is a common challenge in technology innovation and has been named the “valley of death” that can lie between excellent research and the manufacture and commercial success of a product (EARTO 2014).

Thirdly, our research demonstrated how stakeholder engagement and a form of co-design and co-production of knowledge (White and van Koten 2016) could facilitate not only more rapid TRL progression but also less iteration. Interview respondents recognised that involving stakeholders in early discussion of technologies was important, although they also highlighted the challenges of engaging before a prototype was ready. Marzano et al. (2018) concluded that stakeholder engagement not only improved technology design but also enhanced future uptake and strengthened relationships across researchers and other stakeholders that then benefited additional areas of plant health. It has previously been suggested that user-centric and interdisciplinary approaches are effective EARTO (2014) and innovation should be considered within a wider societal context (Yakamura et al. 2013).

Fourthly, this research identified specific attributes of technology innovation within environmental as opposed to other sectors. There are differences between the often intermittent, project based funding for areas such as early detection of pests and the very substantial, long term funding for “missions” such as in space, aviation and defence industries. In the latter, technologies required will be funded and brought to development with some certainty (Mankin 2009); such sectors are more about *how* than *if* technologies are developed. In the public sector, in areas such as health, energy and biosecurity, there are additional challenges. Whilst technologies may meet wider societal and public needs, they may be competing against other emerging technologies (wave energy—Weber 2012) or be struggling to collectively tackle the complexity of the system (composite recycling, in which many different types of composites create multiple diverse technical challenges—Rybicka et al. 2016). Our study also highlights the hybridity of the organisations and partnerships developing the technologies we need for early detection of tree pests and pathogens. Heslop et al. (2001) noted distinct differences between public sector technology development, in universities and research institutions, that tended to focus on “long-term radical innovation processes”, whereas private sector had more explicit success criteria and more closely evaluated product marketing. In the private sector, final development of a technology to commercial product is uncertain (Weber 2012). However, we found that the boundaries across public/private are now diffuse, with hybrid public/private/third sector organisations undertaking much of the research and implementation of tree health (see also White et al. 2018). Biosecurity also currently sits in an ambiguous position across public and private sectors, without the closed system goal of an assured high-technology output. Although there is environmental imperative and policy support for tree health support (Dandy et al 2017), the current era of austerity (White et al. 2018) is creating even more budget restraints than experienced in the early days of LAMP. The shift towards more non-state input to tree health means that responsibility for early detection is being further diffused (Marzano et al. 2018). Hence, we need to ensure that technology innovation is strategically supported across tree health networks. This context means that we now need a mechanism to assist decision makers, funders and investors in identifying and resourcing the development of suitable technologies and we argue that the TRL framework can play a critical role in this effort. This research raises wider questions around green neoliberalism and valuing nature (e.g. Castree 2011).

A further aspect of technology innovation in environmental areas is the need to address complex socio-ecological challenges and not only single issue problems. We may not need the type of closed Systems Readiness Level scheme suggested by Sauser et al (2006), but the framing offered by the MLP (see Yakamura et al. 2013) enables a deeper analysis of our research results within the context of socio-ecological technological innovation (White and van Koten 2016; Marzano et al. 2018). Hence, we can view individual technologies as progressing through TRLs and receiving sufficient investment when the niche innovation (for the particular technology) is active and the socio-technical landscape is permissive. In the case of early detection of tree pests, the socio-technical landscape included cost, policy and stakeholder views. The impacts and societal concern deriving from tree health issues such as Dothistroma Needle Blight, *Phytophthora ramorum*, Asian Longhorn Beetle *(Anoplophora glabripennis),* Oak Processionary Moth (*Thaumetopoea processionea*) and Ash dieback (*Hymenoscyphus fraxineus*), has provoked the release of funds for research and development and catalysed policy that supports a more robust response to the threat of tree pests and pathogens (Dandy et al 2017; Marzano et al 2018). However, we need to continue to actively assess this socio-technical landscape, because technology deployment success is dependent on wider context. For example, a highly effective diagnostic test for tuberculosis failed to achieve anticipated benefits because of poor health care infrastructure in developing countries (Calloway 2017). Health organisations recognised that inserting a new tool into a dysfunctional health system would not be a “game changer” and that investment in the system itself was required. Equally we need to retain a robust system for the early detection of pests, including sufficient well trained inspectors, effective legislation and education and networking with other stakeholders.

## Conclusions

We have demonstrated that a TRL framework can promote the innovation and development of appropriate technologies to detect tree pests and pathogens and facilitate wider biosecurity goals. In particular, it can support continuity of effort and overcome the gap between proof of concept and product commercialisation. Biosecurity management is occurring now within a public/private space in which technologies require economic feasibility and some commercialisation, but where the public good of protecting trees and forests is not fully understood. Thus, the costs and benefits of technology deployment are difficult to calculate. However, technologies alone will not address environmental issues and the TRL framework can help us better understand the process of technology innovation and identify where and when we might invest funding; and when and how we might engage end users and other stakeholders in technology development and utilisation; and if there is ambiguity created by policy surrounding potential deployment. Hence, we need to be cognisant of the wider socio-ecological technical system (Marzano et al 2018). There is no spacecraft in which we can escape the effects of tree pests, but the use of the TRL framework within a socio-ecological technical innovation context (White and van Koten 2016) can support development of individual tools and integrated suites of technologies to enhance stakeholder engagement and better protect our trees, forests and woodlands. It can also encourage us to consider wider societal and political questions such as who is responsible for environmental integrity, and how we invest public and private resources to ensure a sustainable future.

## Supplementary Information

Below is the link to the electronic supplementary material.

Supplementary file1 (PDF 140 kb)

## Footnotes

1 Project title: New approaches for the early detection of tree health pests and pathogens. http://protectingtreehealth.org.uk/. Funded in the UK through the LWEC Tree Health and Plant Biosecurity Initiative (THAPBI).

2 https://aries.ucsd.edu/ARIES/MEETINGS/0712/Waganer/TRL%20Calc%20Ver%202_2.xls

3 https://esto.nasa.gov/files/TRL_Worksheet_11-30-10.xls

4 (downloaded using the following link: files.masscec.com/innovate-clean-energy/NYSERDA-TRLCalculator.xlsm).
