# Supplementary material for: Technology development for the early detection of plant pests: a framework for assessing Technology Readiness Levels (TRLs) in environmental science
Source: J Plant Dis Prot (2006). 2022 Jun 20;129(5):1249–61. doi: 10.1007/s41348-022-00599-3 (PMC9468069; doi:10.1007/s41348-022-00599-3)
Supplement: Supplementary file 1 — Supplementary file1 (PDF 140 kb) [file 41348_2022_599_MOESM1_ESM.pdf]

Supplementary data

Technology development for the early detection of plant pests: a framework for assessing Technology Readiness Levels (TRLs) in environmental science

Rehema White[a], Mariella Marzano[b], Elena Fesenko[c], Alan Inman[c], Glyn Jones[c], Barbara Agstner[c], Rick Mumford[c, 1]

[a] School of Geography and Sustainable Development |University of St Andrews | Irvine Building |North Street |Fife KY16 9AL | SCOTLAND | Tel: +44(0) 1334 462022 | email: rehema.white@st-andrews.ac.uk

[b] Forest Research, Northern Research Station, Roslin, Midlothian, Scotland, EH25 9SY.

[c] Fera Science Ltd, National Agri-Food Innovation Campus, Sand Hutton, York, YO41 1LZ.

[1] Present address: Food Standards Agency, Foss House, Kings Pool, 1-2 Peasholme Green, York YO1 7PR

More detailed narrative of technology development of LAMP

Retrospective application of TRLs to technology development process: The GENIE and the LAMP

The development of on-site detection technologies for use in plant health began with lateral flow devices (LFDs) in the mid to late 1990s (Mumford et al. 2016). These were antibody-based technologies that made use of platforms and chemistries developed, for example, for home-based pregnancy tests. Plant-health-related LFDs reached deployment quickly in the UK as they were easy to use, cheap and fitted easily into the working practices of Plant Health and Seeds Inspectors (PHSI) in what is now the UK's Animal and Plant Health Agency (APHA). LFDs are still extensively used by APHA for on-site screening of plant material.

However, LFDs can lack sensitivity and specificity; for example, the LFD for *Phytophthora* is only genus - and not species - specific. Newer DNA-based diagnostic methods such as the polymerase chain reaction (PCR), widely used now in diagnostic laboratories, have since offered opportunities for increased sensitivity and specificity of on-site detection. Initial proof-

of-concept research showed that a portable PCR machine (the Cepheid Smart Cycler) could be used outside the laboratory to detect plant pests and pathogens. The Cepheid Smart Cycler was a well-developed platform and in military use before being extended to plant health, hence deployment occurred within two years of research beginning on it. The UK was the first country to use this platform in the field for plant health (Tomlinson *et al.*, 2005).

The Smart-Cycler research promoted further understanding of end-user requirements and identified key constraints to deployment by inspectors, namely: (a) high costs, (b) complex DNA extraction steps (c) cross-contamination problems. This led to research on other platforms and chemistries, resulting in the selection of a more-robust and suitable chemistry for field use (Loop- Mediated Isothermal Amplification: LAMP) linked with a more-portable, cheaper and user-friendly platform (the Genie machine from Optisense). The Genie and LAMP system reached the point of deployment in mid-2015 with trained inspectors making front-line diagnostic decisions (without recourse to laboratory confirmation) at a licensed facility at Heathrow airport for *Liriomyza spp.* leafminers on plants imported from South America.

Between 2006 and 2015, the Genie and Lamp technologies were developed using around 20 different funding routes. The research occurred simultaneously with the evolution from a Genie prototype platform to the Genie-I, Genie-II and now the Genie-III machine. This exemplifies the value of working in partnership with SME's with an applied market focus and route to commercialisation. Funding came primarily from longer-term EU projects plus short and longer-term funding sources from the UK government. Mapping their contribution to TRL progress paints a complex picture as the technology was considered for a number of different applications as it was being developed. Thus, its progress can be viewed as that of a technology with a potentially wide application (to multiple plant pests and disease). Key steps and issues that were specifically taken for Genie and LAMP technology development are highlighted in Table 1. Although they generally followed step-wise progression, some steps and projects overlapped or ran in parallel. However, development phases can be approximately aligned with TRL concepts.

The Genie and LAMP technology was thus built on previous research and deployment of on-site technologies (e.g. antibody-based LFDs and DNA-based Smart Cycler platforms) that have moved diagnostics out of the laboratory and into the field. Although the Genie and its isothermal LAMP technology are part of an evolution in on-site diagnostics, there is also a range of novel impacts and benefits for plant health, both now and potentially in the future (e.g.

paving the way for future detection platforms and chemistries that address the next evolution of operation need). These arise mostly from increased affordability (e.g. reduced costs of Genie compared to the Cepheid Smart Cycler) and usability (e.g. smaller size, battery powered and portable, user-friendly interface, closed system preventing cross-contamination). The Genie platform reached potential deployment within three years, but actual deployment was delayed for a further three years due to lack of governance and decision-making processes and because the large number of funding sources created inefficiencies along the pipeline. This highlights the importance of early and effective engagement and long-term capacity building with policy-makers, frontline regulators, industry and other stakeholders; ensuring that 'solutions' are fit-for-purpose, there are routes to commercialisation and they offer a genuine cost benefit. For wider impact, particularly in the field of plant health, there should also be plans for technology transfer to other potential end-users nationally and internationally (e.g. pre- and post-border). In these contexts, using a TRL framework can inform investment decisions and help visualise roadmaps to deployment as well as ensuring that there is continuity provided by a lead organisation or developer. In addition, thinking through TRLs should help identify cost-effective approaches to technology development, such as reducing the time it takes to reach each development stage.

References

Boyd IL, Freer-Smith PH, Gilligan CA, Godfray HCJ (2013) The consequences of tree pests and diseases for ecosystem services. Science 342 (6160): 1235773.

Brasier, C.M. (2008). The biosecurity threat to the UK and global environment from international trade in plants. Plant Pathology 57:792-808.

Britt BL, Berry MW, Browne M, Merrell MA, Kolpack J (2008) Document Classification Techniques for Automated Technology Readiness Level Analysis. J Am Soc Inf Sci Techn 59(4):675–680

Brockerhoff EG, Bain J, Kimberley M, Knížek M (2006) Interception frequency of exotic bark and ambrosia beetles (Coleoptera: Scolytinae) and relationship with establishment in New Zealand and worldwide. Can J For Res 36:289–298.

Bryman A (2001) Social Research Methods. Oxford University Press Centre, Oxford.

Callaway E (2017) Improved diagnostics fail to halt the rise of tuberculosis TB remains a big killer despite the development of a better test for detecting the disease. Nature 551 (7681), 424-425. doi:10.1038/nature.2017.23000

Castree, N. (2011). Neoliberalism and the biophysical environment 3: putting theory into practice. Geography Compass: 35-49. https://doi.org/10.1111/j.1749-8198.2010.00406.x

Clausing D, Holmes M. (2010). Technology Readiness. Research - Technol Management. 53(4):52-59.

Dandy N, Marzano M, Porth E, Urquhart J, Potter C (2017) Who has a stake in ash dieback? A conceptual framework for the identification and categorisation of tree health stakeholders. In: Vasaitis R, Enderle R (eds) Dieback of European Ash (Fraxinus spp.): Consequences and Guidelines for Sustainable Management. Swedish University of Agricultural Sciences, pp 15 – 26.

EARTO (2014) The TRL scale as a research and innovation policy tool. European Association of Research and Technology Organisations Recommendations.

Freer-Smith P, Webber J (2015) Tree pests and diseases: the threat to biodiversity and delivery of ecosystem services. Biodivers Conserv. DOI: 10.1007/s10531-015-1019-0.

GAO (2001) Joint Strike Fighter Acquisition—Mature Critical Technologies Needed to Reduce Risks. GAO-02-39.

Geels FW (2010). Ontologies, socio-technical transitions (to sustainability), and the multi-level perspective. Res Policy 39: 495-510.

Graettinger CP, Garcia S, Siviy J, Schenk RJ, Van Syckle PJ (2002) Using the Technology Readiness Levels scale to support technology management in the DoD's ATD/STO Environments. A finding and recommendations report for Army CECOM. https://resources.sei.cmu.edu/asset_files/SpecialReport/2002_003_001_13931.pdf. Accessed 1 December 2017

Hesplop LA, McGregor E, Griffith M (2001) Development of a Technology Readiness Assessment Measure: The Cloverleaf Model of Technology Transfer. J Technol Transfer 26:369-384.

ISO 16290 (2013) Space systems - Definition of the Technology Readiness Levels (TRLs) and their criteria of assessment, Technical Committee: ISO/TC 20/SC 14, 2013.

Klapwijk MJ, Hopkins AJM, Eriksson L, Pettersson M, Schroeder M, Lindelöw Å, Rönnberg J, Keskitalo ECH, Kenis M (2016) Reducing the risk of invasive forest pests and pathogens: Combining legislation, targeted management and public awareness. Ambio 45 (2): 223-234

Liebhold AM, Brockerhoff EG, Garret LJ, Parke JL, O Britton K (2012) Live plant imports: the major pathway for forest insect and pathogen invasions of the US. Frontier Ecol Environ 10(3):135-143.

Mankins, J.C. (2009). Technology readiness assessments: A retrospective. Acta Astronaut 65: 1216 - 1223.

Marzano M, Allen W, Dandy N, Haight R, Holmes T, Keskitalo ECH, Langer ER, Shadbolt M, Urquhart J (2017) The role of the social sciences in understanding and informing tree biosecurity policy and planning: a global synthesis**.** Biol Invasions 19(11): 3317-3312.

Marzano M, White R, Jones G (2018) Enhancing socio-technological innovation for tree health through stakeholder participation in biosecurity technology development. In: Urquhart J, Potter C, Marzano M (eds) 2018 Human Dimensions of Forest Health. Palgrave Macmillan.

Mumford R, Macarthur A, Boonham N (2016). The role and challenges of new diagnostic technology in plant biosecurity. Food Secur 8: 103-109.

Mumford R, Marzano M, Jones G, White RM (2017). Earlier detection of tree diseases. Impact 7:47-49. **DOI:** https://doi.org/10.21820/23987073.2017.7.47

Pluess T, Cannon R, Jarošík V, Pergl J, Pyšek P, Bacher S (2012) When are eradication campaigns successful? A test of common assumptions. Biol Invasions 14: 1365-1378

Rockstrom, J., W. Steffen, K. Noone, A. Persson, F. S. Chapin, E. F. Lambin, T. M. Lenton, M. Scheffer, C. Folke, H. J. Schellnhuber, B. Nykvist, C. A. de Wit, T. Hughes, S. van der Leeuw, H. Rodhe, S. Sorlin, P. K. Snyder, R. Costanza, U. Svedin, M. Falkenmark, L. Karlberg, R. W. Corell, V. J. Fabry, J. Hansen, B. Walker, D. Liverman, K. Richardson, P. Crutzen and J. A. Foley (2009). A safe operating space for humanity. Nature 461(7263): 472-475.

Rybicka J, Tiwari A, Leeke GA (2016) Technology readiness level of composites recycling technologies. J Clean Prod 112:1001-1012.

Sadin SR, Povinelli F, Rosen R (1989). The NASA technology push towards future space missions. Acta Austronaut 20: 73-77.

Sauser B, Ramirez-Marquez J, Verma D, Gove R (2006) From TRL to SRL: The concept of Systems Readiness Levels. Conference on Systems Engineering Research, Los Angeles, CA, April 7-8.

Straub J (2015) In search of technology readiness level (TRL) 10. Aerosp SciTechnol 46:312-320.

Webber J (2012) WEC Technology Readiness and Performance Matrix – finding the best research technology development trajectory. 4th International Conference on Ocean Energy, 17 October, Dublin. pp1-10

Webber J (2010) Pest risk analysis and invasion pathways for plant pathogens. New Zealand. J For Sci 40 suppl.:45-56.

White, R.M., van Koten, H., 2016. Co-designing for sustainability: strategising community carbon emission reduction through socio-ecological innovation. The Design Journal 19, 25-46.

White RM, Young JC, Marzano M, Leahy S (2018) Prioritising stakeholder engagement for forest health, across spatial, temporal and governance scales, in an era of austerity. J For Ecol and Manag. 417: 313-322

Yakamura H, Kajikawa Y, Suzuki S (2013) Multi-level perspectives with technology readiness measures for aviation innovation. Sustain Sci (2013) 8:87–101 DOI 10.1007/s11625-012-0187-z
